# Then and Now: Reminiscent

**Published:** 1899-01

**Authors:** D. M. Reagan

**Affiliations:** Kyle, Texas


### Then and Now: Reminiscent.

BY D. M. REAGAN, M. D., KYLE, TEXAS.

Read at Austin District Medical Society, December 22, 1898.

We are engaged in a common cause, which calls forth the noblest qualities of mind and heart,—a cause in which many of the brightest intellects, profoundest thinkers and investigators the world has known have spent their long and useful lives. Their memory will be cherished for all time, for the inestimable service they have conferred upon the human race and for the unmeasured good they have accomplished in mitigating human suffering.

I regret that I feel myself so incompetent to present such a matter as you have a right to expect, in order to add my mite to the common stock of wisdom and professional learning deposited in, and worthy of a place in, the archives of this association, or to assist in advancing the object this society so nobly and constantly endeavors to achieve.

In what I have to say, I shall confine myself mainly to recalling a few incidents and reminiscences of the last forty years, in which I have been connected with the profession of medicine and surgery, and in contrasting methods then in vogue with those of to-day.

In attempting to do this, I do not expect to present anything new to the older members of this society, who, like myself, began the laborious life of a country doctor a half a century ago; but it may serve to amuse and interest the younger members for the moment, and if I succeed in doing that, it is all that I can expect in this very imperfect paper.

The facilities for acquiring a medical and surgical education have been increased since the earliest days of medical knowledge, but probably never so rapidly as during the last half century.

We are amazed when we take into account the discoveries in medicine and their adaptation to the relief and cure of diseases, and the wonderful inventions and appliances within our reach, brought into use in the art of surgery within this period.

A half a century ago, physicians were as honest, and worked as hard as now, and with comparatively very few of the physical comforts which the average country doctor enjoys at the present time, and which cheer and lighten in a degree his laborious and too often thankless task. His rides were long and tedious, over rough roads by day and by night, and small fees had to satisfy him. As an illustration of the small reward of the country doctor in a back town and one which to-day would be considered so inadequate for the service, let me relate an experience of Dr. J. B. Frontman, who practiced medicine in my town, Geneva, Alabama, fifty years ago:

One September night a storm, accompanied with a terrific rain storm and wind piled the pine trees so thick across the roads that it was almost impossible to travel, and for three days and nights Dr. Frontman visited all his patients. He received in these three days one dollar in cash, and his total charge was ten dollars for his entire work.

What would my young medical friends present think of such hardship and such insignificant remuneration for three days of such work in professional duties in this year of grace eighteen hundred and ninety-eight?

The average country doctor was poor as well as his patrons, and the only way was to work hard and live economically, and at death leave little or nothing to his family.

Now it is pleasant to know fees are larger, services more fully appreciated, there are less poor and worthless bills, good ones are paid more promptly, and financial conditions are vastly improved (that is, such was the case before the single gold standard was made a law).

During the first ten years of my practice, I did not receive but five dollars for any obstetric case, and in many cases had to wait from twelve months to several years for my pay.

Fifty cents for the first mile, and a shilling for each additional miles, were the charges fifty years ago. The country doctor in those days did nearly all that part of dentistry which consisted in extracting troublesome teeth, the fee for which was nine pence when paid at the time; if "charged," that was usually the end of it. An old doctor once told me that he "pulled," as he called it, over one thousand teeth in one year, with the old-fashioned cant-hook, that abominable instrument of torture,—and if he had not been so much like the immortal "George," who could not tell a lie, I should be tempted to think he lied—exaggerated at least.

I used to think the nine penny fee was due to the pulled rather than the puller, when the pain of extracting was taken into account, and I did not so much blame the patient, after all, for not paying for the torture he suffered in the operation.

A few doctors in olden times made great progress in the art of medicine and surgery, and many discoveries and inventions were brought to the notice of the profession, but the investigators were not numerous in those days, compared with the number now. Money was hard to get, and the average country doctor could not afford the expenses of buying many books to better inform himself in medicine, or instruments and appliances for treating surgical cases. Now conditions are all changed for the better.

There is more money to pay the doctor than formerly, which enables him to buy books, instruments, and whatever is necessary for the successful prosecution of the practice of medical and surgical art; he is able to dress better, ride in better conveyances, live in better houses, and with the increasing of the world, to enjoy life in a much greater degree than in olden times, and feel that he may bequeath something to those he leaves behind him when he departs this life.

The drinking habit was somewhat common in the days gone by, and physicians had great temptation to indulge in that form of dissipation. I regret to say, that within my own remembrance some of the brightest minds in the profession went out in *darkness* from that habit, before the meridian of life was reached. Now, so far as my observation goes, the habitual drunkard in our ranks is the exception to the rule, but not the habitual drinker, i. e., *toper*.

In former times, physicians gave very large doses, and in very crude form, compared with the remedies that are now used. As I remember, the lancet, the cant-hook for teeth, rhubarb, ipecac, epsom salts, calomel, opium, tartar emetic, jalap, gentian root, senna leaves, anise seed, lunar caustic, chamomile flowers, Dovers powders, carbonate of soda, tartaric acid, lead plasters, Burgundy pitch, gum-guaiac, gum-kino, laudanum, paregoric, James powders, and squirting cucumber constituted the principal stock in trade of the country doctor, with the addition of Spanish flies for blistering. Usually, the first thing to do when called to a patient was to bleed, then give a dose of calomel and jalap, and if no operation of the bowels followed in a certain time, an enema was given by means of the pen-end of a goose quill tied within the neck of an inflated hog's bladder, which was used for that purpose, as syringes were scarce in those days, the injection being repeated until the object was accomplished.

I never gave one in that way, but saw it done once by an old doctor of my aqcuaintance.

